# Artificial intelligence-assisted neuropharmacology: reshaping drug discovery and translational strategies for neurological diseases

**DOI:** 10.55730/1300-0144.6176

**Published:** 2026-01-10

**Authors:** Priyanka RATHEE, Sunidhi LOHAN, Sarita KHATKAR, Neelam MALİK, Esra KÜPELİ AKKOL, Renu SEHRAWAT, Pooja RATHEE, Anurag KHATKAR

**Affiliations:** 1 Geeta Institute of Pharmacy, Geeta University, Panipat, Haryana, India; 2 Department of Pharmaceutical Sciences, Guru Jambheshwar University, Hisar, Haryana, India; 3 Department of Pharmacognosy, Faculty of Pharmacy, Gazi University, Ankara, Turkiye; 4 SGT College of Pharmacy, SGT University, Gurugram, Haryana, India; 5 Department of Pharmaceutical Sciences, Maharshi Dayanand University, Rohtak, Haryana, India

**Keywords:** Neurological disorders, digital age, artificial intelligence, machine learning, drug discovery, drug design

## Abstract

The rapid expansion of data-driven technologies, particularly machine learning (ML) and artificial intelligence (AI), has substantially influenced biomedical research and drug discovery. In neuropharmacology, the availability of large-scale genomic, proteomic, chemical, and clinical datasets has stimulated the adoption of AI-based approaches to address persistent challenges in neurological drug development, including high attrition rates and the scarcity of disease-modifying therapies. Unlike prior reviews that broadly discuss AI applications in drug discovery or neurology, this narrative review focuses specifically on neuropharmacology, with an emphasis on translational relevance, disease-oriented examples, and real-world constraints. We critically examine the application of AI and ML across key stages of the neuropharmacological drug discovery pipeline, including target identification, drug–target interaction prediction, lead optimization, toxicity assessment, and early-stage clinical translation. Particular attention is given to concrete case studies in neurodegenerative and neurological disorders, illustrating where AI has meaningfully enhanced discovery efficiency and where its anticipated “revolutionary” impact has not yet been realized. In parallel, we analyze the biological, technical, and regulatory barriers that limit the clinical success of AI-driven strategies, including data bias, limited model interpretability, incomplete understanding of brain biology, and translational bottlenecks. By integrating case-based evidence with a critical analytical perspective, this review delineates both the opportunities and limitations of AI in neuropharmacology. We argue that AI is most effective when deployed as a complementary tool alongside mechanistic neuroscience and clinical expertise, rather than as a standalone solution. As AI methodologies continue to mature, their careful, transparent, and ethically governed integration into neuropharmacological research may advance precision medicine and help bridge persistent gaps in the treatment of neurological disorders.

## 1. Introduction

Neuropharmacology is an exciting field that studies how medications affect the nervous system to alter physiological and pathological processes [1]. Despite significant advancements in our understanding of the pathways driving neurological disorders such as depression, epilepsy, Parkinson’s disease (PD), and Alzheimer’s disease (AD), the development of successful pharmacological treatments remains challenging. Neuropharmacology is the scientific investigation of how drugs alter brain activity and behavior through interactions with receptors, signaling pathways, and neurotransmitters such as gamma-aminobutyric acid, serotonin, and dopamine, which play key roles in regulating mood, cognition, activity, and other physiological functions [2]. Medications targeting enzymes, ion channels, neurotransmitter systems, and other cellular targets are used to treat and alleviate the manifestations of neurological disorders [3]. Neurological conditions are highly complex and heterogeneous, reflecting the interplay of biological, environmental, and physiological factors. Consequently, elucidating the underlying disease mechanisms and identifying appropriate therapeutic targets remain challenging. The blood–brain barrier (BBB) restricts the passage of many medications and therapeutic agents into the brain, thereby reducing the effectiveness of pharmacological treatments for neurological conditions. One of the biggest challenges in drug development is creating medications that can cross the blood–brain barrier while remaining safe and effective [4]. Preclinical drug discovery methods, such as animal studies and in vitro assays, frequently fall short of accurately representing the complex nature of neurological disorders in humans. There is little correlation between preclinical findings and clinical trial outcomes, contributing to a high attrition rate during the therapeutic development process [5]. Many neurological conditions continue to lack adequate treatments, despite recent advances in basic neuroscience research. Although many currently available treatments alleviate symptoms, they do not address the underlying disease pathology or slow disease progression. Neuropharmacological agents can induce a range of side effects, including sedation, movement disorders, cognitive decline, and psychiatric symptoms [6,7]. The development of medications for neurological conditions faces a major challenge in minimizing adverse effects while balancing therapeutic efficacy with tolerability. Finding novel therapeutic uses for currently available medications with well-established safety profiles is known as drug repurposing. Combining drugs to simultaneously target multiple pathways or cellular mechanisms has been shown to enhance therapeutic efficacy and reduce the likelihood of resistance development [8]. With personalized treatment plans, therapeutic outcomes can be maximized while adverse effects are minimized in neurological disorders. Precision medicine aims to tailor treatment strategies for individual patients by integrating genetic background, biomarker profiles, and clinical characteristics [9]. Although neuropharmacology is central to the advancement of therapies for neurological disorders, substantial obstacles remain before key research findings can be translated into clinically effective treatments. To address these challenges, a multifaceted approach integrating translational medicine, pharmacology, neurology, and drug delivery research is required. By adopting cutting-edge technologies, multidisciplinary research models, and personalized treatment approaches, neuropharmacology may help overcome barriers to drug discovery and improve the quality of life of individuals with neurological disorders.

With a focus on the development of novel therapies for neurological disorders, this article critically examines the potential significance of artificial intelligence (AI) in neuropharmacology. Specifically, the review addresses data extraction, target identification, therapeutic design, biomarker discovery, and clinical trial optimization across key stages of drug development. The review aims to provide an in-depth understanding of the current state of AI applications in neuropharmacology. It highlights the potential benefits of AI in neuropharmacology, including improved therapeutic outcomes, reduced costs, increased efficiency, and enhanced accuracy. The review also addresses the major challenges and limitations associated with current therapeutic approaches to neurological disorders. This review aims to foster a balanced and critical discussion of the potential implications of AI for neuropharmacology. The review seeks to advance understanding of the impact of AI on neuropharmacology and its potential to contribute to the identification of novel treatments for neurological disorders.

## 2. Materials and methods

This study adopts a narrative literature review approach to examine the application of AI and machine learning (ML) in neuropharmacology and drug discovery. Rather than aiming for exhaustive coverage, the review emphasizes conceptual understanding, thematic synthesis, and critical analysis of emerging trends, challenges, and translational implications.

Relevant literature published between 2013 and 2025 was identified through systematic searches of major scientific databases, including PubMed, Web of Science, Scopus, and Google Scholar. The search strategy employed combinations of keywords related to neurological disorders, artificial intelligence, machine learning, digital technologies, and drug discovery and design. Articles were selected based on scientific relevance, methodological rigor, and their contribution to understanding AI-driven approaches in neuropharmacology.

Retrieved records were screened using predefined inclusion and exclusion criteria, with priority given to peer-reviewed studies addressing biological data analysis, drug–target interaction prediction, lead optimization, and translational or clinical relevance. The qualitative synthesis highlights both the capabilities and limitations of current AI and ML applications, with particular attention to data quality, model interpretability, and biological validation.

This review distills key AI-driven strategies from a drug discovery perspective, including large-scale biological data integration, predictive modeling of drug–target interactions, and accelerated therapeutic design. The analysis aims to elucidate how AI may reshape treatment strategies for neurological disorders, identify gaps in the existing research landscape, and inform future directions for both experimental and clinical research.

## 3. Harnessing artificial intelligence in drug discovery: a paradigm shift

AI has emerged as a transformative technology in drug discovery for neurological conditions, with the potential to substantially reshape existing discovery and development paradigms. AI has considerable potential to accelerate the identification and development of new treatments, addressing unmet clinical needs in drug discovery [10]. Artificial intelligence refers to the development of computer systems capable of performing tasks—such as learning, problem-solving, and decision-making—that typically require human intelligence [11]. AI plays an increasingly important role in drug discovery by leveraging data-driven methods to predict biological activities, analyze large-scale datasets, and accelerate the development of new therapeutics. Within AI, machine learning involves training algorithms to identify patterns and predict outcomes from large datasets without explicit task-specific programming [12]. Deep learning is an advanced subset of machine learning that learns hierarchical representations of data through multilayered artificial neural networks [13]. In drug discovery, AI has become an influential approach, offering new methods to identify and develop novel therapeutics more efficiently. Deep learning models, machine learning algorithms, and other data-driven strategies constitute key components of AI and collectively influence multiple stages of the drug discovery process. By leveraging AI-driven methodologies, researchers may overcome traditional barriers in drug discovery and open new avenues for pharmaceutical research [14–16]. These advances may create new opportunities to accelerate innovation, tailor therapeutic strategies, improve patient outcomes, and enhance healthcare delivery. Machine learning algorithms enable the analysis of large volumes of chemical and biological data, facilitating the prediction of key features and supporting decision-making in drug discovery. Machine learning algorithms enable rapid screening of large chemical databases to identify high-affinity drug candidates by predicting the likelihood of compound–target protein interactions. Automated learning algorithms that associate chemical structures with biological activities enable the prioritization of lead compounds with favorable pharmacological properties for further experimental validation. Machine learning models can predict potential toxicity, aiding early identification of safety concerns and guiding the selection of compounds with favorable safety profiles [17,18]. Artificial neural networks with multiple layers can identify complex patterns and features within high-dimensional datasets. This capability leads to improved predictive performance and provides new insights into drug discovery processes. Drug candidates can be designed more effectively and selectively using generative algorithms such as variational autoencoders (VAEs) and generative adversarial networks (GANs), which enable the generation of novel chemical structures with desired properties. Convolutional neural networks (CNNs) can predict protein–ligand interactions, identify putative drug targets, and assess compound activity by analyzing images derived from high-throughput screening assays or structural biology data. By analyzing biological sequences such as DNA, RNA, and proteins, recurrent neural networks and long short-term memory networks can infer structure–activity relationships, identify disease-associated mutations, and predict functional properties [19,20]. Data-driven strategies leverage large chemical, biological, and clinical databases to support decision-making and generate novel insights into drug discovery. Integration of multiomics data—including genomics, transcriptomics, and proteomics—provides comprehensive insights into disease mechanisms and drug responses, thereby enabling targeted patient stratification, biomarker discovery, and therapeutic target identification. Machine learning algorithms analyze preliminary data from high-throughput screening assays to identify compounds with the desired biological activity. This process facilitates the analysis of structure–activity relationships, hit identification, and lead optimization. Analysis of real-world data sources, such as patient registries and electronic medical records, enables drug repurposing, pharmacovigilance, and outcomes research by revealing patterns in patient outcomes, treatment responses, and adverse events [21,22].

### 3.1. Applications of artificial intelligence in neuropharmacology: unraveling neurological mechanisms

AI-based drug discovery and clinical neuropharmacology are linked through a translational continuum encompassing target identification, lead optimization, preclinical validation, clinical trials, and therapeutic application. Artificial intelligence plays an important role in early drug development by integrating large-scale genomics and disease network data to improve target selection, although its clinical relevance may be constrained by incomplete mechanistic alignment with disease phenotypes.

AI can further accelerate lead optimization through structure-based virtual screening, pharmacokinetic prediction, and early toxicity assessment, thereby helping to reduce late-stage attrition. In clinical development, AI may enhance trial design, supports patient stratification, and advance precision neuropharmacology, while challenges related to clinical validation and real-world applicability persist.

Rather than replacing clinical expertise, AI complements clinical neuropharmacology by supporting evidence-based drug selection, dosing strategies, and safety monitoring. Clinical interpretation remains essential to ensure that AI-derived insights translate into meaningful patient benefit. Effective integration into clinical decision support systems will depend on explainable and transparent models, training using real-world clinical datasets, and robust regulatory frameworks for AI-assisted prescribing [23–26].

**Figure 1:**
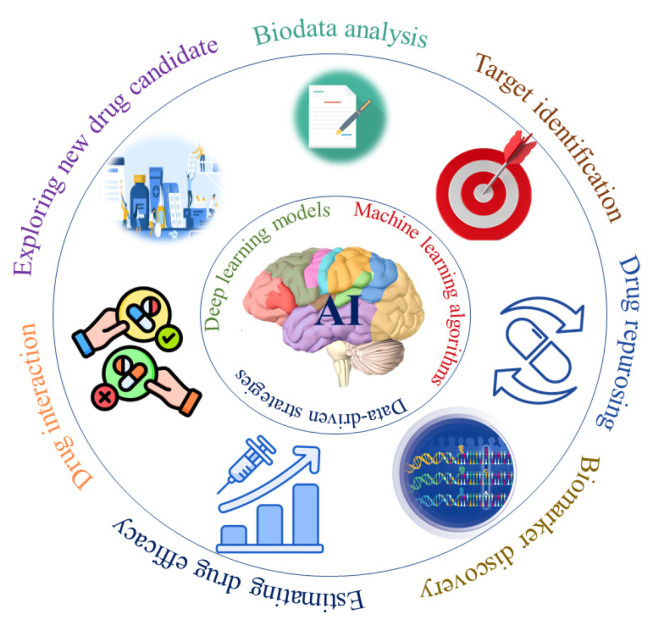
Applications of AI in neuropharmacology.

Overall, AI-driven neuropharmacology represents an important shift in drug discovery and therapeutic development. By enabling biomarker discovery, drug repurposing, and targeted intervention strategies, AI is contributing to advances in the understanding and treatment of neurological disorders (Figure 1).

#### 3.1.1. Target identification and validation

Identifying viable therapeutic targets within the complex networks of biological processes underlying neurological disorders remains a major challenge in drug discovery. AI algorithms, particularly machine learning models, excel at extracting insights from large-scale datasets to identify potential drug targets. These computational approaches identify molecular signatures associated with disease pathophysiology by analyzing proteomic, metabolomic, and genomic data. Moreover, AI-powered approaches facilitate target validation by integrating diverse biological datasets and estimating functional relevance within disease mechanisms, thereby streamlining the transition from target identification to drug discovery [26].

#### 3.1.2. Lead compound identification and optimization

Filtering large chemical databases to identify lead compounds with therapeutic potential is a common practice in conventional drug development. Through machine learning–powered virtual screening methods, AI can automate this process. These algorithms facilitate the identification of lead compounds with favorable drug-like properties by predicting the pharmacokinetic profiles and binding affinities of small molecules. AI-driven approaches also enable the design of novel molecules with improved pharmacological properties, supporting the optimization of lead compounds for neurological disorders [27,28].

#### 3.1.3. Drug repurposing

AI may enable the repurposing of existing drugs for novel therapeutic indications, thereby accelerating the drug discovery process. Machine learning algorithms identify potential repurposing candidates with established pharmacokinetic and safety profiles by analyzing pharmacological databases and large-scale drug–target interaction networks. By bypassing prolonged preclinical testing stages typical of conventional drug development, repurposed medications can be more rapidly evaluated in experimental studies for neurological disorders. Furthermore, AI-based prioritization of drug repurposing candidates can provide insights into potential pathways and therapeutic synergies [29,30].

#### 3.1.4. Estimation of drug efficacy and safety

Accurate prediction of the efficacy and safety of drug candidates is essential for optimizing therapeutic strategies and minimizing adverse effects. AI techniques, including machine learning and deep learning, utilize diverse data inputs to develop predictive models of drug action and toxicity profiles. By integrating clinical, genetic, and molecular data, these models can identify patients at increased risk of complications and predict responses to pharmacological therapy. In addition, AI-driven predictions can support the optimization of therapeutic approaches by enabling individualized dosing regimens tailored to each patient’s unique profile [31,32].

#### 3.1.5. Biomarker discovery and patient stratification

The discovery of biomarkers for neurological disorders is crucial for early detection, prognosis, and treatment planning. AI techniques, such as deep learning, enable the identification of biomarkers associated with disease progression and treatment response through the analysis of omics and neuroimaging data. By supporting the development of noninvasive diagnostic tools and individualized treatment plans, these biomarkers assist clinicians in selecting the most appropriate therapeutic strategies for each patient. AI-driven biomarker discovery also enhances understanding of disease complexity and facilitates the development of precise treatment strategies for neurological disorders [33–35].

#### 3.1.6. Biological data analysis

Data derived from multiple disciplines, including proteomics, genomics, transcriptomics, metabolomics, and structural biology, are collectively referred to as biological data. These complex datasets are analyzed and interpreted using AI techniques to elucidate disease mechanisms, identify potential therapeutic targets, and characterize drug responses. For example, AI systems can process genomic data to identify disease-associated genetic variants, predict gene transcription patterns, and uncover cellular processes involved in disease etiology. Similarly, AI models can analyze transcriptomic data to identify differential gene expression patterns, regulatory networks, and diagnostic signatures associated with disease progression or treatment response [36,37].

#### 3.1.7. Prediction of drug–target interactions

AI methods are applied to predict how pharmaceuticals interact with biological targets, including enzymes, proteins, receptors, and genetic material. Accurate estimation of drug–target interactions is essential for understanding drug mechanisms of action, identifying adverse effects, and prioritizing lead compounds for further development. Machine learning algorithms can learn from large datasets comprising molecular fingerprints, chemical structures, and drug–target interaction profiles. Moreover, AI-assisted techniques such as molecular docking and molecular dynamics simulations enable atomic-level modeling of drug–target protein interactions. By predicting binding affinity, binding modes, and the structural stability of drug–target complexes, these computational techniques support rational drug design by providing detailed insights into drug–target interactions [38,39].

#### 3.1.8. Discovery of novel drug candidates

AI techniques can be employed to identify novel drug candidates with favorable pharmacological properties, such as potency, selectivity, and safety. AI models identify and rank potential drug candidates for clinical validation by leveraging extensive chemical databases, structural information, and biological data. Various generative models, including variational autoencoders, generative adversarial networks, and deep reinforcement learning approaches, can generate novel chemical structures with predefined attributes. By sampling new molecules that optimize key drug-like parameters such as bioavailability, solubility, and synthetic accessibility, these models can explore the underlying distribution of chemical space. AI-driven virtual screening techniques screen large chemical libraries to identify compounds with high affinity for target proteins or relevant biological pathways. Machine learning algorithms prioritize compounds with a high likelihood of biological activity based on analyses of physicochemical properties, molecular fingerprints, and biochemical descriptors [40–42].

## 4. Neurological disorders and challenges in modern therapy

Neurological disorders encompass a wide range of conditions affecting the nervous system, including the brain, spinal cord, and peripheral nerves. These disorders can be broadly categorized into neurodegenerative diseases, such as Alzheimer’s and Parkinson’s disease, cerebrovascular diseases such as stroke, and other conditions, including epilepsy and multiple sclerosis, which are characterized by progressive dysfunction of neurons in the brain and spinal cord. Despite significant advances in medical science, treating neurological disorders remains a complex and challenging endeavor. An overview of three common neurological conditions is provided below.

### 4.1. Alzheimer’s disease

Alzheimer’s disease (AD) is a neurodegenerative disorder that progressively worsens over time. The disorder is characterized by progressive decline in memory and cognitive functions, accompanied by difficulties in performing everyday activities. Among older adults, AD represents the most common cause of dementia. A key pathological feature of AD is the accumulation of aberrant protein aggregates in the brain, including beta–amyloid plaques and tau neurofibrillary tangles, which contribute to neuronal dysfunction and cell death. Clinical manifestations typically begin with mild symptoms and progressively worsen over time. These symptoms eventually progress to severe cognitive impairment, behavioral disturbances, and loss of functional independence. At present, AD has no curative treatment; available therapies primarily aim to alleviate symptoms and slow disease progression. Pharmacological interventions, such as memantine and cholinesterase inhibitors, are commonly used to improve cognitive function and mitigate cognitive decline [43,44].

### 4.2. Parkinson’s disease

Parkinson’s disease (PD) is a chronic neurodegenerative disorder characterized by rigidity, postural instability, bradykinesia, and tremor. The pathological hallmark of PD is the degeneration of dopaminergic neurons in the substantia nigra, leading to dopamine deficiency and dysfunction of motor control circuits. In addition to motor symptoms, PD is associated with a range of nonmotor manifestations, including mood disturbances, autonomic dysfunction, sleep disorders, and cognitive decline. PD treatment regimens are designed to alleviate symptoms and improve quality of life. Pharmacological treatments include anticholinergic agents, monoamine oxidase B inhibitors, and dopamine replacement therapies, such as levodopa and dopamine agonists. Patients with refractory or advanced disease may benefit from surgical interventions such as deep brain stimulation [45,46].

### 4.3. Epilepsy

Epilepsy is a chronic neurological disorder characterized by recurrent unprovoked seizures resulting from abnormal electrical activity in the brain. Epilepsy may arise from diverse etiologies, including genetic factors, traumatic brain injury, central nervous system infections, structural brain abnormalities, and metabolic disorders. Epilepsy is managed using therapy, lifestyle modifications, and, in selected cases, surgical interventions. The cornerstone of treatment is antiseizure medication (also referred to as antiepileptic drugs), which is tailored to the individual patient based on seizure type, frequency, and clinical characteristics [47,48]. Globally, millions of individuals are affected by neurological disorders such as epilepsy, PD, and AD. To improve treatment outcomes and quality of life, continued research is required to deepen understanding of the underlying mechanisms driving these conditions and to develop more effective therapies.

The complexity of the nervous system, the heterogeneity of neurological diseases, and challenges in effective treatment delivery make these disorders formidable challenges in contemporary medicine. Despite symptomatic relief provided by existing medications, novel strategies targeting the underlying causes of neurological disorders are urgently needed. Advances in neuroimaging, gene therapy, stem cell research, neuroprotective agents, precision medicine, and AI may help overcome these challenges and improve outcomes for patients with neurological disorders. By leveraging these advanced tools and methodologies, researchers and clinicians may move closer to developing transformative therapies that meaningfully improve the lives of individuals with neurological disorders. The human nervous system is highly complex due to its extensive networks of neuronal and glial cells. This complexity makes it difficult to identify precise therapeutic targets and to predict the effects of different treatment strategies [49]. The blood–brain barrier (BBB) serves as an effective defense against external threats but also restricts the passage of many therapeutic agents into the brain. One of the major challenges in treating neurological disorders is developing medications that can effectively penetrate the blood–brain barrier without causing systemic toxicity [4]. The underlying pathophysiology, disease course, and clinical manifestations of neurological disorders are often highly heterogeneous. This variability complicates the development of universally effective therapies and necessitates individualized treatment approaches. For many neurological disorders, reliable biomarkers for diagnosis, disease progression, and treatment response are lacking. The absence of such biomarkers hampers accurate assessment of treatment efficacy and limits opportunities for early intervention. Many medications currently used to treat neurological disorders are associated with significant adverse effects, which can limit their long-term effectiveness. As novel therapies are developed, maintaining an appropriate balance between safety and efficacy remains crucial. Because neurological conditions often progress slowly, extended study durations are required to evaluate the efficacy and safety of novel treatments. Consequently, clinical investigations become more costly, time-consuming, and complex. Ethical considerations are particularly important in neurological research and clinical trials, especially when involving vulnerable populations such as individuals with cognitive impairment. Regulatory requirements further complicate the development and approval of innovative therapies [50–53].

## 5. Limitations of current therapeutic approaches in neurological disorders

Although many patients experience symptomatic relief from current treatment approaches for neurological conditions, these approaches frequently have substantial limitations. These limitations underscore the critical need for novel therapeutic strategies that improve patient outcomes while addressing the complex etiology of these conditions. Many current therapies focus primarily on symptom management rather than addressing the underlying causes of disease. Although these therapies may provide temporary symptom relief, they do not alter disease course or halt disease progression. This limitation is particularly evident in neurodegenerative diseases such as Parkinson’s and Alzheimer’s disease, for which no disease-modifying therapies are currently available to halt neurodegeneration or meaningfully delay disease progression, offering only modest symptomatic benefit [54]. The effectiveness of many pharmacological treatments for neurological disorders is limited, with substantial interindividual variability in patient response. These medications may also be associated with significant adverse effects and poor tolerability, leading to treatment discontinuation or reduced adherence. For example, the use of antipsychotic medications for behavioral symptoms in dementia is associated with an increased risk of adverse effects, including sedation, extrapyramidal symptoms, and metabolic disturbances [55]. Despite significant advances in neuroscience research, the underlying mechanisms of many neurological disorders remain incompletely understood. This limited understanding hampers the development of targeted therapies capable of effectively modifying disease processes. The development of disease-modifying treatments is hampered, in part, by an incomplete understanding of the precise mechanisms underlying the accumulation of tau neurofibrillary tangles and beta-amyloid plaques in AD [56]. A major challenge to effective drug delivery to the central nervous system (CNS) is posed by the blood–brain barrier. The BBB restricts the delivery of many therapeutic agents, including biologics and small molecules, thereby limiting their clinical efficacy in treating neurological disorders. Consequently, the development of novel drug delivery strategies to enhance CNS penetration is imperative and represents a substantial challenge for the pharmaceutical industry [57,58]. The clinical presentation, disease course, and therapeutic outcomes of neurological disorders frequently exhibit substantial variability. This variability—arising from differences in disease phenotype, genetic background, comorbidities, and environmental factors—complicates therapeutic decision-making. To maximize treatment efficacy and minimize adverse effects, personalized medicine strategies that account for this heterogeneity are required [59]. In light of these limitations, novel therapies capable of overcoming current barriers and providing more effective treatments for neurological disorders are urgently needed. Innovative drug delivery systems, targeted biologics, gene-based therapies, and disease-modifying agents represent promising approaches for addressing the underlying pathophysiology of neurological disorders and improving patient outcomes. Achieving personalized and effective therapies for neurological disorders will require multidisciplinary collaboration, advances in biomarker discovery, and the integration of precision healthcare initiatives.

## 6. AI-driven methodologies in neuropharmacology

AI-driven approaches and techniques in neuropharmacology have substantially transformed the drug discovery process, enabling more efficient identification and optimization of novel treatments for neurological disorders. AI-driven methods such as molecular docking, quantitative structure–activity relationship (QSAR) modeling, and virtual screening play a key role in accelerating drug development [60]. By leveraging machine learning algorithms and computational approaches, researchers can efficiently analyze large chemical libraries, predict ligand–target interactions, and optimize lead compounds with improved pharmacological properties for the treatment of neurological disorders. Virtual screening is a computational approach for identifying potential drug candidates from large chemical libraries without the need for extensive experimental screening. AI-driven virtual screening methods employ machine learning algorithms to predict a compound’s likelihood of binding to a specific protein based on its molecular structure and physicochemical properties. ML models trained on data from established ligand–target interactions acquire the ability to identify binding patterns and features, which can then be used to rank ligands with the highest predicted binding affinities for subsequent experimental validation [61–63]. Molecular docking is a computational method used to predict atomic-level binding interactions between ligands and target protein receptors. AI-driven molecular docking algorithms employ optimization techniques and machine learning models to estimate energetically favorable ligand–protein complex conformations and binding modes. Machine learning models trained on known ligand–protein interaction data enable the prioritization of lead compounds for further experimental investigation. These models are trained to assess ligand binding affinity and specificity for target proteins of interest. Molecular docking algorithms frequently incorporate scoring functions to evaluate the quality of predicted ligand binding poses. These scoring functions rank compounds according to their predicted binding propensity and likelihood of interaction with the target protein [62,64–66]. QSAR modeling enables computational prediction of the biological activity of chemical compounds based on their structural features and physicochemical properties. AI-powered QSAR models use machine learning algorithms to quantitatively correlate chemical descriptors with biological activity data. Machine learning models trained on large datasets of chemical compounds and associated biological outcomes learn relationships between molecular descriptors and biological endpoints. QSAR models can be used to predict the pharmacological properties of novel compounds, optimize the therapeutic efficacy and specificity of lead compounds, and prioritize candidates based on their predicted biological activity profiles prior to experimental validation [67,68].

**Figure 2:**
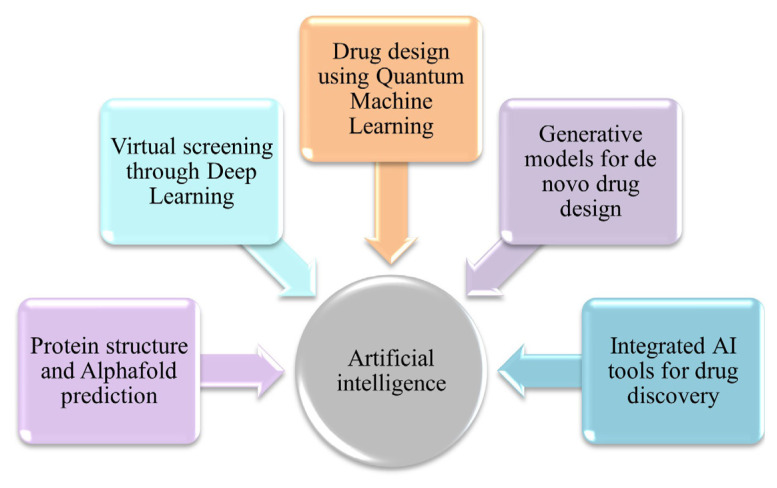
Selected examples of successful AI applications in the identification of potential drug candidates.

In recent years, several practical applications of AI have been demonstrated in the identification of potential therapeutics for neurological disorders, highlighting the transformative impact of AI-driven approaches and their adaptability and effectiveness in neuropharmacology. As AI technologies continue to advance, they have the potential to significantly accelerate the discovery and development of innovative treatments addressing unmet medical needs in neurology. Figure 2 summarizes selected notable examples of successful AI applications in identifying potential drug candidates for neurological conditions. First, AlphaFold (Google DeepMind, London, United Kingdom), an AI-driven protein structure prediction system, has substantially advanced the field of protein structure prediction, particularly for proteins associated with neurological disorders. By accurately predicting the three-dimensional structures of proteins implicated in neurological disorders such as PD and AD, AlphaFold facilitates the rational design of small molecules and biologics with improved precision and selectivity [69,70]. In addition, deep learning-based virtual screening techniques have been effectively applied to identify potential therapeutic candidates for neurological disorders. For example, Atomwise (Atomwise Inc., San Francisco, CA, USA), a prominent AI-driven drug discovery company, screened millions of small molecules using deep learning algorithms to identify inhibitors of the sigma-1 receptor, a protein target implicated in neurodegenerative diseases. This effort led to the identification of novel small-molecule inhibitors with potential therapeutic relevance for conditions such as amyotrophic lateral sclerosis (ALS) and AD [71,72]. More recently, quantum machine learning has accelerated the discovery of novel drugs for neurological disorders by enabling the simulation of molecular interactions and prediction of chemical properties. Using classical machine-learning algorithms, researchers have designed novel small molecules targeting protein–protein interactions associated with neurological disorders. These computational approaches leverage features of quantum computing technology to optimize lead compounds with desirable therapeutic properties and to model complex molecular systems [73,74]. In addition, generative algorithms such as variational autoencoders (VAEs) and generative adversarial networks (GANs) have been used to generate novel chemical structures with potential therapeutic relevance for neurological disorders. Generative models were employed by In silico Medicine (In silico Medicine, Hong Kong, China) a biotechnology company specializing in AI-driven drug discovery, to develop novel small molecules targeting specific proteins implicated in neurodegenerative diseases. Following synthesis and experimental validation, these AI-generated compounds demonstrated efficacy in preclinical models of neurological disorders [75,76]. Researchers from academia, industry, and clinical medicine collaborate through shared AI platforms—such as the Mila–Quebec AI Institute’s MoleculeNet—to advance AI-driven drug discovery for neurological diseases. MoleculeNet supports simulation-based screening, molecular docking, QSAR modeling, and related drug discovery tasks by integrating diverse AI approaches and computational techniques. Using this platform, researchers can prioritize compounds for further experimental validation in neurological disease models, identify promising drug candidates, and estimate their therapeutic potential [77].

## 7. Case studies

The complexity of the human brain and the multifactorial nature of neurological disorders present substantial challenges for conventional drug discovery. AI-driven methodologies, which can process large-scale datasets, identify latent patterns, and predict biological outcomes, offer new opportunities to overcome these barriers. In neuropharmacology, AI has demonstrated value across target identification, compound screening, drug repurposing, biomarker discovery, and patient stratification, thereby contributing to the acceleration of disease-modifying therapy development. Selected case studies illustrating both the translational potential and current limitations of AI-based approaches in neurological drug discovery are summarized in Table.

### 7.1. AI-driven case analyses in neuropharmacology

#### 7.1.1. Case 1: AlphaFold-enabled structure-based drug design in Alzheimer’s and Parkinson’s diseases

AlphaFold has substantially advanced target validation in neurodegenerative drug discovery by providing high-confidence structural predictions for proteins that are experimentally challenging to resolve, including amyloid-β and tau aggregates, presenilin-1, APOE4, α-synuclein, LRRK2, and DJ-1. These targets were prioritized based on their central roles in pathogenic cascades such as protein misfolding, aggregation, and kinase dysregulation.

AlphaFold-derived structures were integrated into structure-based virtual screening pipelines, combining molecular docking with machine learning–based scoring functions and QSAR models to prioritize compounds with high predicted affinity and favorable safety profiles. Most programs remain at the in silico or early preclinical stage, with candidate refinement supported by molecular dynamics simulations, biophysical assays, and neuronal cell models.

Preclinical studies report increased hit rates and more rational ligand selection compared with traditional screening approaches. Several tau-targeting compounds have demonstrated reduced aggregation and behavioral improvement in transgenic mouse models; however, convincing evidence of disease-modifying efficacy in humans remains lacking, and clinical translation is still under evaluation [69,70].

#### 7.1.2. Case 2: Atomwise deep learning–based virtual screening for sigma-1 receptor–mediated neuroprotection

Atomwise employed convolutional neural networks (CNNs) to identify novel ligands targeting the sigma-1 receptor, a protein implicated in neuroprotection, calcium homeostasis, endoplasmic reticulum (ER) stress regulation, and neuronal survival. CNN models trained on three-dimensional representations of protein–ligand complexes were used to screen millions of compounds, followed by medicinal chemistry–driven optimization to improve potency, selectivity, blood–brain barrier penetration, and metabolic stability.

Lead compounds progressed through preclinical validation, demonstrating nanomolar binding affinity, favorable pharmacokinetic profiles, and neuroprotective effects in cellular models of ALS and oxidative stress. Despite encouraging in vitro and in vivo findings, these programs remain at the preclinical stage, and clinical efficacy in neurodegenerative diseases has yet to be established [71,72].

#### 7.1.3. Case 3: Insilico medicine–driven generative design of neurodegenerative drug candidates

Insilico Medicine applied generative adversarial networks (GANs) and variational autoencoders (VAEs) to design novel small molecules targeting proteins implicated in synaptic dysfunction, proteostasis, autophagy, and neuroinflammation. Targets such as GSK-3β, Bcl-2 family proteins, and immune regulators were prioritized using network-based disease biology and chemogenomic analysis.

Generative models trained on extensive chemical libraries generated novel scaffolds optimized simultaneously for target potency, CNS penetration, and pharmacokinetic properties. Several AI-designed compounds advanced to preclinical testing, demonstrating favorable binding profiles and neuroprotective effects in cellular and animal models of AD and ALS. Nonetheless, these candidates remain in early development, and progression to clinical evaluation continues to face well-recognized challenges inherent to CNS drug development [75,76].

#### 7.1.4. Case 4: AI-assisted imaging biomarker discovery in epilepsy and multiple sclerosis

Beyond small-molecule discovery, AI has been leveraged to identify imaging biomarkers that are critical for disease stratification and clinical trial optimization. In epilepsy, machine learning models trained on MRI-derived morphometric features have achieved high diagnostic accuracy for temporal lobe epilepsy and enabled localization of disease-relevant structural abnormalities. In multiple sclerosis, deep learning applied to FLAIR MRI has been used to predict disability progression and lesion evolution, thereby facilitating early identification of aggressive disease phenotypes.

These AI-derived biomarkers are currently in the clinical validation phase, with retrospective studies demonstrating improved diagnostic and prognostic performance. Although prospective multicenter validation is ongoing, clinical deployment remains limited, and these tools currently function as adjuncts rather than replacements for expert clinical interpretation [78–80].

## 8. Critical and analytical perspectives

Neurological disorders—including AD, PD, epilepsy, depression, schizophrenia, and multiple sclerosis—represent a major global health burden due to their biological complexity, clinical heterogeneity, and limited therapeutic success. Traditional neuropharmacology has largely focused on symptomatic relief and trial-and-error drug discovery rather than disease modification. In this context, artificial intelligence has emerged as a potentially transformative tool, offering unprecedented capacity for large-scale data integration, pattern recognition, and predictive modeling.

AI-driven neuropharmacology integrates machine learning, deep learning, natural language processing, and big-data analytics to process complex datasets spanning genomics, proteomics, neuroimaging, electrophysiology, and electronic health records. Unlike reductionist, single-target approaches, AI enables systems-level analyses that capture multitarget interactions and network-based disease mechanisms, conceptually aligning with the multifactorial nature of neurological disorders. Through gene–disease network analysis, virtual screening of large chemical libraries, and optimization of lead compounds for neurotoxicity and blood–brain barrier permeability, AI has contributed to accelerating early-stage drug discovery while reducing cost and time.

However, the predictive power of AI models is fundamentally constrained by the quality of the underlying data. Incomplete understanding of brain biology, biased or homogeneous datasets, and limited representation of diverse patient populations can lead to misleading or overly optimistic predictions [105]. Although AI enables personalized neuropharmacology through the integration of genetic profiles, neuroimaging biomarkers, and patient-specific drug response data, clinical implementation remains limited by infrastructure requirements, cost, and ethical concerns related to data privacy and informed consent.

AI models can predict drug–drug interactions, neurotoxicity, and cognitive or behavioral adverse effects over time, yet they often fail to capture the full biological and clinical complexity of neurological diseases. Regulatory authorities remain cautious, particularly given the limited explainability and transparency of many high-risk AI applications. AI-based analysis of magnetic resonance imaging (MRI), positron emission tomography (PET), and electroencephalography (EEG) data has improved early diagnosis and treatment monitoring; however, excessive reliance on algorithmic outputs risks diminishing clinical judgment and increasing dependence on proprietary technologies.

Key challenges in AI-powered neuropharmacology include algorithmic bias, black-box model opacity, concerns related to patient privacy and data security, and regulatory uncertainty. Without robust ethical frameworks and effective oversight, AI may exacerbate healthcare inequities and undermine patient trust. While AI has the potential to reshape neurotherapeutics through accelerated development, improved safety prediction, and individualized treatment strategies, its success depends on high-quality data, ethical governance, enhanced model interpretability, and rigorous regulatory integration. Ultimately, a balanced and critical integration of AI with traditional neuropharmacology is essential to realize its therapeutic potential while safeguarding patient welfare [102–105].

### 8.1. Real-world challenges

Despite widespread enthusiasm, the real-world impact of AI-driven drug discovery in neuropharmacology has been uneven. Neurological diseases pose exceptional challenges due to the complexity of the central nervous system, blood–brain barrier constraints, and incompletely understood disease mechanisms. Although AI has improved early-stage discovery—such as target identification through gene–disease networks, lead optimization, virtual screening, neurotoxicity prediction, and drug repurposing—these advances have not translated into proportional clinical success. Most AI-assisted drug candidates continue to fail before or during clinical trials [106].

In AD research, AI has been extensively applied to tau- and amyloid-related targets as well as drug repurposing efforts. However, many AI-guided candidates have failed to demonstrate meaningful cognitive benefit, often reinforcing existing hypotheses rather than uncovering novel disease mechanisms. This limitation reflects training on historically biased datasets that perpetuate incomplete or flawed disease models. Similarly, AI-based repurposing efforts identified several non-CNS drugs with apparent neuroprotective potential; however, many failed due to poor BBB penetration, inadequate efficacy in animal models, or unexpected CNS toxicity—highlighting the gap between computational prediction and biological reality.

Large-scale pharmaceutical AI initiatives have yielded early promise but limited long-term progress in neurology. Key barriers include overreliance on curated datasets, challenges in integrating heterogeneous clinical and omics data, and limited interpretability of model outputs. Consequently, AI predictions often require substantial human intervention, thereby reducing their standalone utility [107]. The prevalence of black-box models further complicates regulatory approval, clinician adoption, and reproducibility, positioning AI primarily as a decision-support tool rather than an autonomous decision-maker.

Despite significant investment, AI has not yet substantially reduced development timelines or costs for CNS drugs. Instead, it has shifted the field toward data-driven discovery, which must complement—rather than replace—mechanistic neuroscience and clinical expertise. AI shows clear value in toxicity prediction, lead optimization, biomarker discovery, and patient stratification, but remains limited in causal inference and long-term outcome prediction.

A hybrid framework combining explainable AI with mechanistic neuropharmacology and rigorous biological validation is therefore essential. Future success will depend on improved CNS-specific datasets, multimodal data integration, and advances in transparent AI models, alongside parallel progress in fundamental neuroscience. At present, AI’s contribution to neuropharmacology appears evolutionary rather than revolutionary. While early-stage discovery has been transformed, meaningful clinical breakthroughs remain scarce, underscoring the need for cautious optimism, ethical oversight, and translational rigor [108,109].

## 9. Challenges and future directions

AI presents substantial potential for addressing challenges across neurological disorders but continues to face hurdles related to data quality, interpretability, generalizability, algorithmic bias, and regulatory barriers. The integrity and accessibility of data constitute major obstacles in AI-driven drug development. Large volumes of high-quality data are required to develop AI models with reliable predictive accuracy. Effective AI model training is challenging because healthcare data are frequently heterogeneous, inconsistent, or incomplete. Furthermore, concerns related to confidentiality and intellectual property rights may restrict access to sensitive data. Understanding the internal structure and decision-making processes of deep learning and other advanced AI models remains challenging. This lack of transparency may hinder acceptance of AI-generated predictions by researchers, clinicians, and regulatory authorities. These stakeholders require clear understanding of how model decisions are generated, particularly in relation to drug efficacy and patient safety. Significant technological and logistical barriers exist to integrating AI into established drug research workflows. Integrating AI tools into traditional drug discovery pipelines necessitates workflow modifications, infrastructure adaptation, and specialized researcher training. A key challenge is ensuring seamless integration without disrupting ongoing research activities. The regulatory framework governing AI applications in drug research is still evolving. Ensuring compliance with emerging AI-specific regulations while adhering to existing regulatory standards remains challenging. Additional challenges arise from ethical concerns such as algorithmic bias, clinical data privacy, and the potential for erroneous AI predictions. Addressing and mitigating algorithmic bias is essential to ensure equitable and effective drug discovery efforts across diverse demographic populations. AI system development and maintenance require substantial investment in data storage infrastructure, computational resources, and specialized expertise. The cost of implementing AI-driven drug discovery approaches may be prohibitive for smaller research institutions and biotechnology companies. Data security and patient confidentiality represent additional ethical concerns that must be carefully addressed.

Promoting partnerships and data-sharing initiatives among academic institutions, pharmaceutical companies, and healthcare organizations is crucial for overcoming data-related obstacles. Anonymized and standardized datasets can improve both the availability and quality of data for AI training. Developing techniques that elucidate AI model decision-making processes can increase trust and facilitate the integration of AI into drug discovery workflows. Transparent and interpretable models are more likely to be accepted by the scientific community and approved by regulatory authorities. The application of AI in drug research requires the development of clear criteria and evidence-based guidelines by regulatory bodies. Robust legal frameworks must be established to ensure that AI-driven drug discovery complies with safety, efficacy, and ethical standards. To develop comprehensive and effective regulatory frameworks, collaboration among regulatory agencies, AI researchers, and pharmaceutical companies is essential [110–112].

Future directions include personalized medicine through AI-driven analysis of individual patient data, real-time monitoring via wearable devices, multimodal data fusion for comprehensive disease understanding, and explainable AI techniques to build trust. In parallel with these developments, AI-enabled neurotechnological approaches are emerging as complementary strategies alongside pharmacological interventions. In this context, Neuralink (Neuralink Corp., Fremont, CA, USA) represents a prominent example of innovative engineering through its AI-powered brain–computer interface (BCI). The neurotechnology startup Neuralink aims to develop BCI systems that enable direct communication between the brain and external devices. These systems use AI-driven decoding based on machine learning models to interpret neural signals. BCIs may serve as an adjunct to pharmacotherapy by offering precise neuromodulation when pharmacological treatments fall short. A major challenge lies in overcoming translational bottlenecks that hinder progression from controlled trials to broader clinical application.

Additionally, explainable AI techniques are pivotal for building trust among clinicians, researchers and patients. Establishing robust regulatory frameworks and fostering collaborative research initiatives are essential for the responsible integration of AI into clinical practice, with the potential to advance the diagnosis, treatment, and management of neurological disorders and improve patient outcomes and healthcare delivery. Interdisciplinary partnerships among neurologists, pharmacologists, regulatory authorities, and computer scientists will be essential for future progress.

## 10. Conclusion

AI represents a paradigm shift in neuropharmacology by offering innovative solutions to address key challenges posed by the complexity of the CNS and the multifactorial nature of neurological disorders. AI holds significant promise for transforming the understanding, diagnosis, and treatment of complex neurological disorders through the application of machine learning algorithms, deep learning models, and data-driven techniques. AI has been incorporated into neuropharmacology to accelerate the processing of large biological datasets, enhance the identification of potential drug candidates beyond conventional methods, and predict drug–target interactions with high precision. Despite this promise, substantial challenges remain in the effective utilization of AI in neuropharmacology. These challenges include navigating regulatory barriers, addressing the opaque nature of many AI models, and ensuring access to large-scale, high-quality datasets while accounting for ethical considerations. Advances in AI technology, when combined with robust regulatory frameworks, have the potential to enable more individualized and effective treatment approaches for neurological disorders. As AI continues to evolve, it may substantially reshape the field of neuropharmacology, particularly in drug discovery. Such developments may offer patients with debilitating neurological disorders renewed optimism and lay the foundation for the implementation of more personalized and efficacious treatment strategies.

## Figures and Tables

**Table:** AI-driven case studies in neurological disorders.

Neurological disorder	Conventional diagnostic methods	AI-driven diagnostic research	Key outcomes of the study	Reference
Epilepsy	Detailed medical history and seizure characterization Electroencephalogram (EEG) Magnetic resonance imaging (MRI) Computed tomography (CT) scan Blood tests Neurological examination	Under the ENIGMA-Epilepsy initiative, this study employs AI and global collaboration to analyze region-of-interest–level MRI data for the diagnosis of temporal lobe epilepsy (TLE). Machine learning techniques are used to differentiate patients with temporal lobe epilepsy (TLE) from healthy control subjects.	AI identifies structural abnormalities associated with TLE through region-of-interest (ROI) analysis. Assists clinicians in complex epilepsy diagnoses. Enhances the accuracy of epilepsy diagnosis and treatment planning. Has the potential to significantly improve healthcare outcomes.	[78]
		Developed a clinical decision support tool integrated with AI-driven EEG analysis for epilepsy diagnosis, utilizing patient EEG data and machine learning techniques.	An AI-powered clinical decision support tool improves the accuracy and efficiency of epilepsy diagnosis. Clinicians report increased diagnostic confidence, faster interpretation, and improved accuracy when supported by AI. Integration of AI streamlines diagnostic workflows, facilitating earlier treatment initiation.	[79]
		The study employed a deep learning approach using convolutional neural networks (CNNs), which are well-suited to image analysis tasks. The CNNs were trained on a dataset comprising MRI scans from individuals with confirmed mesial temporal lobe epilepsy (MTLE) and from control subjects without epilepsy.	A deep learning model demonstrates promising performance in the diagnosis of MTLE. The model achieves high accuracy, sensitivity, and specificity in distinguishing MRI scans of patients with MTLE. CNNs effectively learn discriminative features from neuroimaging data for MTLE diagnosis.	[80]
Multiple sclerosis	Comprehensive medical history and physical examination Diagnostic criteria (e.g., McDonald criteria) Magnetic resonance imaging (MRI) Cerebrospinal fluid (CSF) analysis Evoked potentials Blood tests	The study describes the development and validation of an AI model using fluid-attenuated inversion recovery (FLAIR) MRI data to predict clinical disability in individuals with multiple sclerosis (MS).	Demonstrates the model’s efficacy in accurately predicting disability progression. Identifies critical imaging features correlated with disability. Discusses potential clinical applications. Acknowledges limitations and proposes avenues for future research.	[81]
		In this study, AI is used to develop models for detecting contrast-enhancing lesions in MRI scans of patients with MS. MRI data are collected and annotated, followed by the training and validation of AI models, typically based on CNNs. These models enhance diagnostic accuracy, efficiency, and clinical utility in the diagnosis and monitoring of MS. Key challenges include variability in imaging protocols and the requirement for large, well-annotated datasets.	AI models demonstrate higher sensitivity and specificity compared with traditional methods. Rapid AI-assisted analysis reduces radiologists’ interpretation time. Accurate lesion detection supports early diagnosis, treatment planning, and disease monitoring. Overall, AI enhances the detection of contrast-enhancing lesions in multiple sclerosis MRI scans, benefiting diagnosis and patient management.	[82]
		AI-based MS management using retinal imaging involves the collection of diverse datasets, image preprocessing, training deep learning models to classify MS status, result validation, and integration of the system into clinical practice for diagnosis and monitoring.	Early detection of MS-related changes. Objective disease progression assessments. Personalized treatment insights. Remote patient monitoring facilitated through telemedicine. Research contributions, include the identification of novel biomarkers and disease-associated pathways.	[83]
Parkinson’s disease	Quantitative finger motion analysis Functional neuroimaging techniques Surface electromyography (EMG) analysis Plantar pressure analysis	The system accurately identifies gait patterns associated with PD. The system demonstrates potential for early detection of PD, thereby supporting timely clinical intervention. The system is capable of categorizing disease stages based on gait characteristics.	Demonstrates high accuracy and sensitivity in classification tasks. Shows potential for improving diagnostic accuracy and enabling personalized treatment strategies.	[84]
		In an innovative study, AI was used to remotely assess PD severity. Wearable devices combined with machine learning were used to track real-time symptoms beyond standard clinical evaluations, including movement patterns, tremors, and gait disturbances. The study aimed to provide personalized insights to support early intervention and individualized treatment planning.	Promising results demonstrate methodological feasibility and diagnostic accuracy. AI-enabled home-based assessments are capable of evaluating PD severity. Has the potential to transform patient care and disease management strategies.	[85]
		The tutorial on PD recognition using single-photon emission computed tomography (SPECT) images and interpretable AI employs a multistep methodology. The process begins with data acquisition and preprocessing of SPECT images, followed by feature extraction and model development, typically utilizing a CNN architecture. Interpretable AI techniques, such as Gradient-weighted class activation mapping (Grad-CAM) and Shapley Additive exPlanations (SHAP), are then integrated to provide insights into the model’s decision-making process. Model evaluation involves performance metrics such as accuracy, along with clinical validation.	The methodology effectively identifies PD. Provides clinically interpretable and easily understandable insights. Success in diagnosis and interpretation is affirmed.	[86]
Alzheimer’s disease	Comprehensive medical history and physical examination Cognitive and standardized neuropsychological assessments Laboratory tests Structural neuroimaging (MRI, CT scans) PET scans Clinical diagnostic criteria (DSM, NIA-AA criteria) Assessment of functional status and daily living abilities	The study examining the application of AI in the genetic analysis of AD yielded significant findings. Through AI-based analysis, specific genes were identified as being associated with an increased risk of developing AD, providing insights into the genetic mechanisms underlying the condition. Moreover, predictive models were developed that leverage genetic profiles to enable early detection of AD, potentially facilitating timely intervention and treatment.	AI-based analysis of gene expression patterns uncovers potential therapeutic targets. Identification of genetic markers for personalized treatment approaches. Integration of diverse data sources enhances understanding of the genetic architecture of AD. Demonstrates potential for further advancements in AD research and clinical practice.	[87]
		The Hybrid AI-Based Model for Detection of AD (HTML) employs a multifaceted methodology that integrates multiple AI techniques to enhance disease detection. A comprehensive dataset is collected and preprocessed, with an emphasis on brain morphological features derived from MRI scans. By combining machine learning and deep learning approaches, a hybrid AI model is developed and optimized using ensemble learning strategies.	The model demonstrates superior accuracy compared with traditional methods. Demonstrating robustness and potential clinical utility for early intervention.	[88]
		Advancements in medical imaging and AI have substantially transformed the diagnosis and prognosis of AD. Subtle brain changes and abnormal protein deposition associated with AD can be detected using modalities such as MRI, PET, cerebrospinal fluid (CSF) biomarkers, and AI-based analytical algorithms. AI algorithms analyze these imaging data to identify disease-specific patterns, while trained models predict disease progression and prognosis using combined imaging and clinical information.	Increased accuracy in early diagnosis. Timely interventions and personalized treatment strategies. AI-based prognostic models provide valuable insights into disease progression. Aid in patient management and therapeutic decision-making. Integration of multimodal imaging and AI advances the diagnosis and prognosis of early-stage AD. Improves patient care and outcomes.	[89]
Amyotrophic lateral sclerosis	Comprehensive clinical assessment Electromyography (EMG) Nerve conduction studies (NCS) Neuroimaging studies (MRI or CT scans) Laboratory tests Established clinical diagnostic criteria Multidisciplinary clinical evaluation	The primary finding of the study is the application of AI for multimodal in vivo disease staging in amyotrophic lateral sclerosis (ALS). This approach integrates multiple imaging modalities with clinical data to assess disease progression and severity. AI algorithms analyze MRI data and clinical information to identify patterns and biomarkers associated with ALS progression.	Shows promise for improving diagnostic accuracy in ALS. Offers potential for enhanced prognostic prediction. Holds promise for tailoring personalized treatment strategies for patients with ALS.	[90]
		In a study investigating AI-assisted quantification of hypothalamic atrophy in ALS using CNN-based automatic segmentation, a multistep methodological process was employed. MRI datasets from ALS patients and control subjects were curated, images were preprocessed for quality enhancement and intensity normalization, and a CNN model was subsequently trained using supervised learning. This resulting model, incorporating convolutional and pooling layers, automatically segmented the hypothalamic region. Rigorous evaluation on independent datasets assessed model performance in terms of accuracy, sensitivity, and specificity.	Significantly improved hypothalamic segmentation accuracy was observed. Facilitates precise quantification of hypothalamic atrophy in patients with ALS. Demonstrates potential for early diagnosis and disease progression monitoring.	[91]
		Machine learning algorithms facilitated multiomics data analysis from ALS patients and control subjects, enabling the identification of key molecular pathways associated with the disease. Potential therapeutic targets were prioritized based on their impact on disease-associated pathways and subsequently validated through experimental assays and preclinical models, supporting their relevance in ALS.	Contributes to an improved understanding of ALS pathophysiology. Identification of novel therapeutic targets. Increases optimism for future drug development efforts. Potential for the development of novel therapeutic interventions.	[92]
Migraine	Comprehensive medical history review, Physical examination, International Classification of Headache Disorders (ICHD) criteria, Diagnostic investigations, including blood tests and imaging studies, Prospective headache diary documentation, Referral to appropriate specialists (e.g., neurologist, headache specialist) when indicated.	The study evaluates the accuracy of an AI-based diagnostic system for migraine detection, reporting performance metrics such as sensitivity, specificity, and overall accuracy in comparison with traditional diagnostic methods. The study also discusses limitations, potential clinical applications, and implications for future research.	The study reports performance metrics including sensitivity, specificity, positive predictive value (PPV), negative predictive value (NPV), and overall accuracy of the AI-based system for migraine diagnosis compared with established diagnostic criteria or expert clinical judgment.	[93]
		Machine learning algorithms are trained on labeled datasets derived from electronic health records (EHRs), incorporating features such as clinical notes and diagnostic codes. Natural language processing (NLP) techniques are applied to facilitate structured data extraction. The developed models are validated using standard performance metrics to assess accuracy.	AI enables the development of accurate and scalable tools for automated migraine detection in electronic health records (EHRs). Enables efficient screening, diagnosis, and management. Enhances clinical decision-making and patient outcomes. Facilitates clinical research and trial design.	[94]
		A comprehensive dataset was compiled and meticulously preprocessed to ensure data consistency. Advanced machine learning techniques were employed to identify key features and construct the diagnostic model using state-of-the-art algorithms. Rigorous evaluation and clinical validation were conducted to ensure diagnostic accuracy and clinical relevance.	The AI model accurately diagnoses migraine in pediatric and adolescent populations, outperforming traditional diagnostic approaches. Enables early detection and supports personalized treatment recommendations. Offers potential relief for healthcare systems by reducing diagnostic burden. Advances the application of AI in healthcare for pediatric and adolescent populations. Demonstrates potential for future innovations in diagnosis and treatment.	[95]
Cerebral palsy	Comprehensive assessment by a multidisciplinary healthcare team, including pediatricians, neurologists, and developmental specialists. Medical history, Physical examination, Developmental assessment and neurological examination, Relevant neuroimaging studies.	AI-based technologies have the potential to support monitoring and assessment of motor function progression in children with cerebral palsy, offering a more objective and accurate evaluation compared with traditional methods.	The main finding of the study indicates that AI-based technologies can effectively detect clinically relevant changes in gross motor function in children with cerebral palsy.	[96]
		AI integration follows a structured, methodical approach to streamline gross motor function assessments in children with cerebral palsy. First, a dataset comprising assessment scores and clinical data is collected for model training. Second, a machine learning algorithm, such as a neural network, is trained on these data to identify relevant patterns and correlations. Third, the trained AI model is integrated into the assessment process, supporting clinicians by automatically analyzing data and providing real-time insights.	Implementation of AI in gross motor function assessments for children with cerebral palsy improves efficiency and diagnostic accuracy. Automated scoring and analysis reduce time requirements and resource utilization for clinicians. AI provides data-driven insights derived from extensive datasets, supporting clinical treatment decisions. Early detection capabilities enable timely adjustment of therapeutic interventions. Has the potential to improve overall clinical outcomes for children with cerebral palsy.	[97]
		The AI-based approach for predicting motor function in children and adolescents with cerebral palsy involves the collection of a comprehensive dataset encompassing assistive device usage and patient characteristics. Relevant features are extracted, preprocessed, and input into ML algorithms, such as neural networks or decision trees, to identify relationships between input features and motor outcomes. Iterative model refinement and validation, including techniques such as cross-validation, are employed to ensure model generalizability.	AI-based approaches develop predictive models using assistive device data to accurately estimate gross motor function. These models offer valuable insights for clinicians and caregivers. Tailored interventions and support strategies can be developed based on these insights. Enhances individualized care for individuals with cerebral palsy.	[98]
Huntington’s disease	Comprehensive clinical assessment, Family history assessment, Targeted genetic testing, when indicated.	AI analyzes large-scale datasets that integrate genetic data and clinical records to identify patterns and biomarkers associated with Huntington’s disease. These tools support clinicians in predicting disease progression and tailoring individualized treatment strategies. Integrating AI into Huntington’s disease care has the potential to improve patient outcomes and provide deeper insights into disease mechanisms.	AI applications the diagnosis and management of Huntington’s disease demonstrate promising potential. Enhances diagnostic accuracy. Improves the effectiveness of disease management strategies. Offers meaningful advancements in healthcare for patients with Huntington’s disease.	[99]
		AI-based approaches for genetic risk assessment of Huntington’s disease using facial analysis begin with the compilation of facial image datasets from individuals with and without the causative gene mutation. Machine learning algorithms are trained to identify disease-associated facial features, such as alterations in facial symmetry and expression. The trained models subsequently analyze facial images of individuals with unknown genetic status to estimate the likelihood of carrying the Huntington’s disease–associated gene mutation.	The outcome of this methodology is a noninvasive and potentially cost-effective approach for screening Huntington’s disease. This approach enables earlier detection and intervention for individuals at risk. However, this approach remains in the research phase and requires rigorous validation before widespread implementation in clinical settings.	[100]
		Research on Huntington’s disease symptom modeling employs artificial neural networks (ANN) and fuzzy logic, beginning with the collection of extensive patient data encompassing clinical symptoms, biomarkers, genetic information, and demographic variables. Following data preprocessing and quality assurance, ANN- and fuzzy logic–based models are developed. ANNs capture complex symptom–disease relationships, while fuzzy logic frameworks address uncertainty within clinical data. Through iterative training and validation, these models demonstrate the ability to predict disease-related symptoms with high accuracy.	Reported outcomes include early disease detection, personalized treatment strategies, improved clinical decision support, and enhanced understanding of disease mechanisms.	[101]
Traumatic brain injury	Comprehensive medical history and clinical evaluation, Glasgow Coma Scale, Neurological examination, Computed tomography (CT) scan, Magnetic resonance imaging (MRI), Positron emission tomography (PET) scan Standardized neuropsychological testing, Electroencephalogram (EEG), Evoked potentials.	AI-powered CT analysis has emerged as a pivotal tool for optimizing diagnostic and treatment processes, ultimately contributing to improved outcomes for patients with traumatic brain injury (TBI).	The main finding of the study highlights that AI-driven analysis of CT scans significantly improves clinical care for patients with traumatic brain injury (TBI). By improving efficiency and accuracy, AI algorithms support early detection of intracranial lesions, provide quantitative assessments of injury severity, assist in personalized treatment planning, and integrate into clinical workflows.	[102]
		The article presents an AI-based method for predicting early mortality risk during emergency room triage in patients with TBI. A large retrospective dataset is analyzed using machine-learning techniques such as logistic regression and decision tree models. The models undergo rigorous training and validation using cross-validation, with performance evaluated based on sensitivity, specificity, and AUC-ROC metrics.	The results demonstrated that the AI-based system exhibited promising accuracy and reliability in predicting early mortality risk in patients with TBI during emergency room triage. Integration of such a system into clinical practice could enhance clinical decision-making and improve patient outcomes by enabling timely identification and prioritization of high-risk cases, thereby facilitating prompt intervention and appropriate resource allocation.	[103]
		The study employed a prospective design, enrolling patients presenting to the emergency department with suspected mild traumatic brain injury (mTBI). Upon admission, serum samples were collected and analyzed for apolipoprotein A-I (Apo A-I) and S100 calcium-binding protein B (S100B) levels using standardized assays. Clinical data and CT imaging findings were recorded along with follow-up assessments. Statistical analyses, including ROC curve analysis, were performed to evaluate the diagnostic accuracy of the biomarkers.	Results indicated that both Apo A-I and S100B levels hold promise for the diagnosis of mTBI. Elevated S100B levels correlated with abnormal CT findings, indicating potential utility in predicting intracranial pathology following mTBI. Apo A-I effectively differentiated patients with mTBI from control subjects. These findings suggest that serum biomarkers may enhance mTBI diagnosis and prognostic assessment, thereby supporting targeted clinical interventions.	[104]
